# Gender and survival of critically ill patients: results from the FROG-ICU study

**DOI:** 10.1186/s13613-019-0514-y

**Published:** 2019-03-29

**Authors:** Alexa Hollinger, Etienne Gayat, Elodie Féliot, Catherine Paugam-Burtz, Marie-Céline Fournier, Jacques Duranteau, Jean-Yves Lefrant, Marc Leone, Samir Jaber, Alexandre Mebazaa, Mattia Arrigo, On behalf of the FROG ICU study investigators, Alain Cariou, Nicolas Deye, Jacques Duranteau, Bertrand Guidet, Samir Jaber, Laurent Jacob, Jean-Yves Lefrant, Marc Leone, Qin Lu, Alexandre Mebazaa, Virginie Montiel, Isabelle Rennuit, Emmanuel Samain, Tarek Sharshar, Antoine Tesniere, Antoine Vieillard-Baron, Michel Wolff

**Affiliations:** 1 Department of Anesthesiology, Critical Care and Burn Unit, Hôpitaux Universitaires Saint Louis – Lariboisière, Assistance Publique – Hôpitaux de Paris, Université Paris Diderot - Paris 7, Sorbonne Paris Cité, UMR-S 942, INSERM, Paris, France; 2 Intensive Care Unit, Assistance Publique - Hopitaux de Paris, University Hospital Ambroise Paré, 26930 Boulogne-Billancourt, France; 3 Department of Anesthesia, Surgical Intensive Care, Prehospital Emergency Medicine and Pain Therapy, University Hospital Basel, Basel, Switzerland; 4 Anesthesiology and Perioperative Care Medicine Department, APHP Hopital Beaujon and University, Paris 7, France; 5 Département d’Anesthésie-Réanimation, UMR 942, Hôpitaux universitaires Paris-Sud, Hôpital de Bicêtre, 78, rue du Général Leclerc, 94270 Le Kremlin-Bicêtre, France; 6 Service des Réanimations, CHU Nîmes, Place du Pr Robert Debré, 30029 Nîmes Cedex, France; 7 Department of Anesthesiology and Intensive Care (DAR B), Saint Eloi University Hospital, Montpellier, France; 8 PhyMedExp, INSERM U-1046, CNRS, Montpellier University, Montpellier, France; 9 Department of Cardiology, University Hospital Zurich, Zurich, Switzerland; 10 Department of Anesthesiology and Intensive Care, Saint Louis – Lariboisière University Hospitals, 2 rue Ambroise Paré, 75010 Paris, France; 11 Department of Anaesthesiology and Critical Care Medicine, AP-HM, Hôpital Nord, Marseille, France

**Keywords:** Gender, Female, Women, Critically ill, ICU, Mortality, Outcome

## Abstract

**Purpose:**

Few studies analyzed gender-related outcome differences of critically ill patients and found inconsistent results. This study aimed to test the independent association of gender and long-term survival of ICU patients.

**Materials and methods:**

FROG-ICU was a prospective, observational, multi-center cohort designed to investigate the long-term mortality of critically ill adult patients. The primary endpoint of this study was 1-year mortality after ICU admission of women compared to men.

**Results:**

The study included 2087 patients, 726 women and 1361 men. Women and men had similar baseline characteristics, clinical presentation, and disease severity. No significant difference in 1-year mortality was found between women and men (34.9% vs. 37.9%, *P* = 0.18). After multivariable adjustment, no difference in the hazard of death was observed [HR 0.99 (95% CI 0.77–1.28)]. Similar 1-year survival between women and men was found in a propensity score-matched patient cohort of 506 patients [HR 0.79 (95% CI 0.54–1.14)].

**Conclusion:**

Women constituted one-third of the population of critically ill patients and were unexpectedly similar to men regarding demographic characteristics, clinical presentation, and disease severity and had similar risk of death at 1 year after ICU admission.

*Trial registration* ClinicalTrials.gov NCT01367093; registered on June 6, 2011.

**Electronic supplementary material:**

The online version of this article (10.1186/s13613-019-0514-y) contains supplementary material, which is available to authorized users.

## Introduction

Critically ill patients display in-hospital mortality rates up to 20–40%, [1–3] as well as impaired long-term survival and quality of life [4, 5]. Most ICU trials investigating outcome focused mostly on short-term survival with little regard for long-term outcome and studied the population of critically ill patients as a whole, neglecting potential differences associated with gender.

Few studies analyzed gender-related differences in short- and long-term mortality of ICU patients and found inconsistent results. A large Swedish ICU study showed that male gender was associated with higher consumption of ICU resources and longer ICU stay, but with similar short-term mortality compared to women [6]. On the contrary, male gender was associated with improved in-hospital survival in a study on sepsis and septic shock [7]. Other ICU studies did not find relevant differences in short- and long-term survival between women and men [8, 9].

Even fewer data are available for gender-related differences in long-term survival after critical illness. Moreover, long-term survival is mostly dependent on the burden of comorbidities after ICU discharge, which may vary across genders, as recently described by our group and others [5, 9]. Therefore, whether the gender itself or the associated demographic and clinical characteristics may influence the long-term survival of critically ill patients is unknown.

The primary aim of this French and euRopean Outcome reGistry in Intensive Care Unit (FROG-ICU) sub-study was to test the hypothesis that gender is independently associated with long-term survival of critically ill patients.

## Materials and methods

### Study design

FROG-ICU was a prospective, observational, multi-center cohort designed to investigate long-term mortality of critically ill adult patients. The study was performed in accordance with Good Clinical Practice and the Declaration of Helsinki 2002, validated by the corresponding ethical committees and registered on ClinicalTrials.gov (NCT01367093). Patients were recruited from August 2011 to June 2013.

The study design was published previously [10]. Briefly, all consecutive patients admitted to any of the 28 participating ICUs in 19 hospitals in France and Belgium were screened for eligibility. Inclusion criteria were requirement for invasive mechanical ventilation and/or vasopressor or inotrope drug support for more than 24 h following ICU admission. Exclusion criteria were age less than 18 years, severe head injury, brain death or persistent vegetative state, organ transplantation in the last 12 months and/or lack of social security coverage.

The primary endpoint of this study was 1-year mortality. The secondary endpoint was 28-day mortality.

### Statistical analysis

Continuous variables are expressed as median (interquartile range), and nominal variables are expressed as number (percentages). Differences between independent groups were assessed with Wilcoxon rank sum test, Mann–Whitney *U*-test, and Fisher’s exact test, as appropriate.

Survival was plotted with the Kaplan–Meier curve, and differences between groups were tested with the log-rank test. Unadjusted and covariate-adjusted Cox proportional hazards models were used to evaluate the association between gender and 1-year mortality, resp. 28-day mortality after ICU admission. The relative hazard is expressed as hazard ratio (HR) with 95% confidence interval (CI). Adjustments were performed for Simplified Acute Physiology Score II (SAPS II), Sequential Organ Failure Assessment (SOFA) score, and Charlson Comorbidity Index (CCI). Subgroup analyses were performed for age (below vs. above the median) and diagnosis groups at ICU admission.

To further reduce the bias related to the difference in baseline characteristics between women and men, the primary and secondary endpoints were investigated in a propensity score-matched cohort.

To create a propensity score on gender, gender was explained by the following clinical variables: age, body mass index, SAPS II, Glasgow coma scale, heart rate, mean blood pressure, temperature, pH, hemoglobin, platelets, white blood count, creatinine, urea at inclusion, diagnosis at inclusion (heart failure/cardiogenic shock/sepsis/neurological disease/hemorrhagic shock/trauma/postoperative), Charlson Comorbidity Index, hypertension, dyslipidemia, diabetes mellitus, smoking status, alcohol status, coronary artery disease, valvular heart disease, chronic heart failure, peripheral vascular disease, prior stroke, cognitive dysfunction, loss of autonomy, chronic obstructive pulmonary disease, chronic liver disease, chronic renal disease, renal replacement therapy, malignancy, human immunodeficiency virus infection, acute respiratory insufficiency, tracheostomy. Ratio for matching was to 1 for 1 (one man associated with one women) with a caliper of 20%, using the “nearest neighbor” method. Standardized difference of mean or prevalence between men and women is calculated for each covariate of propensity score to check whether characteristics between groups are well balanced. Matching was accepted when all standardized differences were smaller than 10%. The matching process significantly reduced differences in baseline characteristics (see Additional file 1: Figure 1).

The null hypothesis was rejected with an adjusted two-sided *P* value < 0.05. All statistical analyses were performed using R statistical software (The ”R” Foundation for Statistical Computing, Vienna, Austria).

## Results

### Patient characteristics during ICU stay

**Fig. 1:**
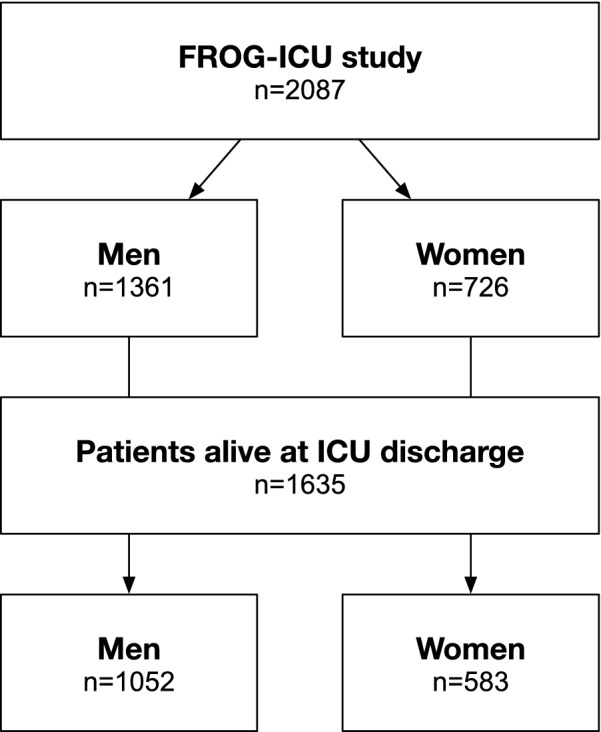
Study population of the FROG-ICU cohort

The study included 2087 patients, 726 women and 1361 men (Fig. 1). Patient characteristics are summarized in Table 1. Women and men had globally similar baseline characteristics (i.e., age, burden of comorbidities, and Charlson Comorbidity Index), similar clinical presentation (i.e., blood pressure, heart rate, use of vasopressors, mechanical ventilation), and similar disease severity, according to SAPS II and SOFA score, as shown in Table 1. Significant differences between women and men were found with respect to some preexisting diseases, ICU referral diagnosis, laboratory parameters at admission, and the need for renal replacement therapy during ICU stay, as shown in Table 1. ICU mortality and length of stay were 21.7% and 12 [7; 21] days, respectively, without relevant differences between women and men, as shown in Table 2.

**Table 1:** Patient characteristics at ICU admission

Patient characteristics	All *N* = 2087	Women *N *= 726	Men *N *= 1361	*P* value*
Demographic data				
Age (year)	63 [51; 74]	63 [51; 74.8]	63 [51; 74]	0.59
Body Mass Index (kg/m2)	26.5 [23.1; 30.8]	26 [22.5; 31.2]	26.6 [23.5; 30.3]	0.51
Comorbidities				
Hypertension	902 (43.3%)	319 (43.9%)	583 (43%)	0.67
Dyslipidaemia	412 (19.8%)	123 (16.9%)	289 (21.3%)	0.017
Diabetes melllitus	384 (18.4%)	121 (16.7%)	263 (19.4%)	0.13
Coronary artery disease	188 (9%)	35 (4.8%)	153 (11.3%)	< 0.001
Valvular heart disease (severe)	82 (3.9%)	31 (4.3%)	51 (3.8%)	0.57
Heart failure	153 (7.3%)	57 (7.9%)	96 (7.1%)	0.52
Peripheral vascular disease	209 (10%)	60 (8.3%)	149 (11%)	0.05
Prior stroke	92 (4.4%)	25 (3.4%)	67 (4.9%)	0.11
COPD	273 (13.1%)	70 (9.6%)	203 (15%)	0.001
Chronic kidney disease	241 (11.6%)	80 (11%)	161 (11.9%)	0.57
Chronic liver disease	158 (7.6%)	52 (7.2%)	106 (7.8%)	0.59
Active malignant tumors	281 (13.5%)	93 (12.8%)	188 (13.9%)	0.51
HIV	53 (2.5%)	12 (1.7%)	41 (3%)	0.06
Loss of autonomy	78 (3.7%)	38 (5.2%)	40 (2.9%)	0.009
Cognitive dysfunction	33 (1.6%)	13 (1.8%)	20 (1.5%)	0.58
Diagnosis at ICU admission				
Respiratory disorder	89 (4.3%)	21 (2.9%)	68 (5%)	0.017
Trauma	536 (25.7%)	182 (25.1%)	354 (26%)	
Sepsis/septic shock	179 (8.6%)	53 (7.3%)	126 (9.3%)	
Neurological disease	286 (13.7%)	120 (16.5%)	166 (12.2%)	
Cardiac arrest	394 (18.9%)	142 (19.6%)	252 (18.5%)	
Cardiogenic shock	146 (7%)	55 (7.6%)	91 (6.7%)	
Hemorrhagic shock	110 (5.3%)	31 (4.3%)	79 (5.8%)	
Planned surgery	165 (7.9%)	52 (7.2%)	113 (8.3%)	
Others	181 (8.7%)	70 (9.6%)	111 (8.2%)	
Vital parameters				
Temperature (°C)	37.2 [36.7; 37.8]	37.2 [36.7; 37.7]	37.3 [36.8; 37.9]	0.001
Mean blood pressure (mmHg)	81.3 [72.3; 92]	81.3 [72.3; 91.7]	81.2 [72.3; 92]	0.81
Heart rate (bpm)	92 [78; 106]	92 [79; 106]	91 [77; 106]	0.60
Glasgow coma scale	14 [5; 15]	14 [4; 15]	14 [5; 15]	0.18
Laboratory values at admission				
Arterial pH	7.4 [7.4; 7.5]	7.4 [7.4; 7.5]	7.4 [7.4; 7.5]	0.12
Lactate (mmol/L)	1.4 [1; 2]	1.4 [1.1; 2]	1.4 [1; 1.9]	0.09
Leukocytes (G/L)	10.9 [7.6; 16.2]	11.6 [8.2; 17]	10.6 [7.4; 15.8]	0.004
Hemoglobin (g/L)	100 [89; 114]	99 [88; 109]	101 [90; 117]	< 0.001
Platelets (G/L)	164 [99; 244]	171 [98; 257]	162 [101; 237]	0.15
BUN or Urea (mg/dL)	8.4 [5.2; 14]	7.1 [4.4; 12.6]	9.1 [5.6; 14.8]	< 0.001
Creatinine (mg/dL)	84 [59; 150]	69 [50; 119]	94 [66; 165]	< 0.001
Bilirubin (umol/L)	13 [8; 27]	11 [7; 21]	14 [9; 29]	< 0.001
Organ support				
Vasopressors at admission	1502 (72.2%)	526 (72.5%)	976 (72%)	0.84
Ventilation during ICU stay:				
Invasive	1632 (80.3%)	561 (79.7%)	1071 (80.6%)	0.76
Non-invasive	316 (15.6%)	115 (16.3%)	201 (15.1%)	
RRT during ICU stay	480 (23%)	137 (18.9%)	343 (25.2%)	0.001
Disease severity scores				
Charlson score	3 [1; 5]	3 [1; 5]	3 [1; 5]	0.15
SAPS II	49 [36; 63]	50 [35; 62]	48 [36; 63]	0.85
SOFA score	8 [5; 10]	7 [5; 10]	8 [5; 11]	0.12

Data presented as median [interquartile range] and *P* value obtained with Mann–Whitney *U*-test for continuous variables and as number (percentage) with Fisher’s exact test for categorical variables

**Table 2:** Patient outcomes in the overall cohort (n=2087)

	All *N* = 2087	Women *N* = 726	Men *N* = 1361	*P* value
ICU length of stay (days)	12 [7; 21]	12 [7; 21]	13 [7; 22]	0.58
ICU mortality	452 (21.7%)	143 (19.7%)	309 (22.7%)	0.11
28-day mortality	443 (21.3%)	139 (19.2%)	304 (22.4%)	0.09
1-year mortality	767 (36.9%)	253 (34.9%)	514 (37.9%)	0.18

Data are presented as number (percentage) or median [interquartile range], as appropriate

### Primary endpoint (1-year mortality)

**Fig. 2:**
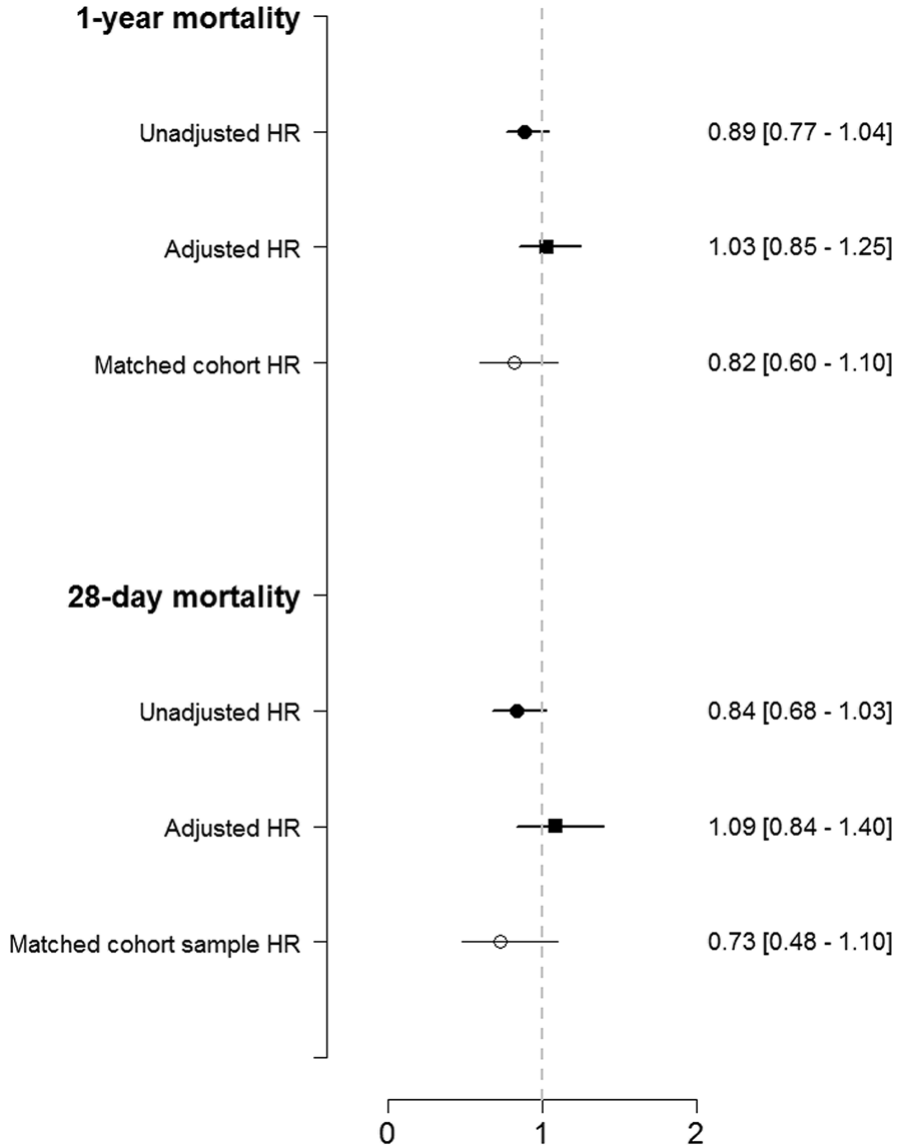
Forest plot for mortality according to gender. The adjustment was performed for SAPS II, SOFA score, and Charlson Comorbidity Index

No significant difference in 1-year mortality after ICU admission (34.9% vs. 37.9%, *P* = 0.18) was found between women and men, as shown in Table 2. Accordingly, there was no difference in the hazard of death for women compared to men [HR 0.87 (95% CI 0.72–1.06)], as shown in Fig. 2. After multivariable adjustment, no difference in the hazard of death was found [HR 0.99 (95% CI 0.77–1.28)], as shown in Fig. 2.

**Fig. 3:**
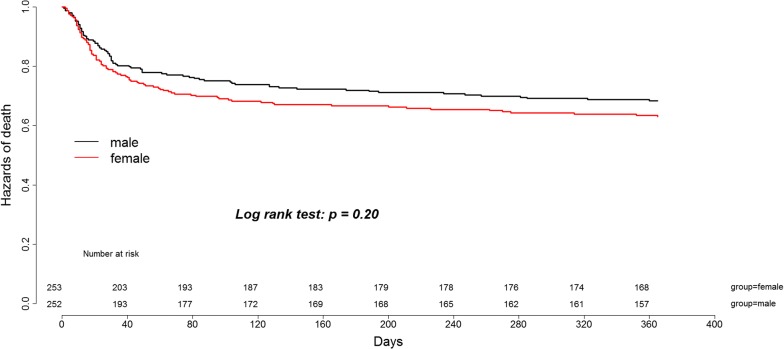
One-year survival of the propensity score-matched cohort

Similar 1-year survival between women and men was found in a propensity score-matched patient cohort of 506 patients (Table 3, Fig. 3). Accordingly, the hazard of death at 1 year after ICU admission was similar for women compared to men in the matched cohort [HR 0.79 (95% CI 0.54–1.14)], as shown in Fig. 2.

**Table 3:** Patient outcomes in the propensity score-matched cohort (*n* = 506)

	All *N* = 506	Women *N* = 253	Men *N* = 253	*P* value
28-day mortality	93 (18.4%)	40 (15.8%)	53 (21%)	0.13
1-year mortality	173 (34.3%)	80 (31.6%)	93 (36.9%)	0.20

Data are presented as number (percentage)

### Secondary endpoint (28-day mortality)

No significant difference in survival was found between women and men at 28 days after ICU admission (19.2% vs. 22.4%, *P* = 0.09), as shown in Table 2. Accordingly, there was no difference in the hazard of death for women compared to men [HR 0.82 (95% CI 0.65–1.02)], as shown in Fig. 2. After multivariable adjustment, no difference in the hazard of death was found [HR 1.08 (95% CI 0.79–1.47)], as shown in Fig. 2.

Consistent results were found in the propensity score-matched cohort of 506 patients (Table 3) with no difference in the hazard of death at 28 days after ICU admission between women and men [HR 0.70 (95% CI 0.44–1.11)], as shown in Fig. 2.

### Subgroup analysis

**Fig. 4:**
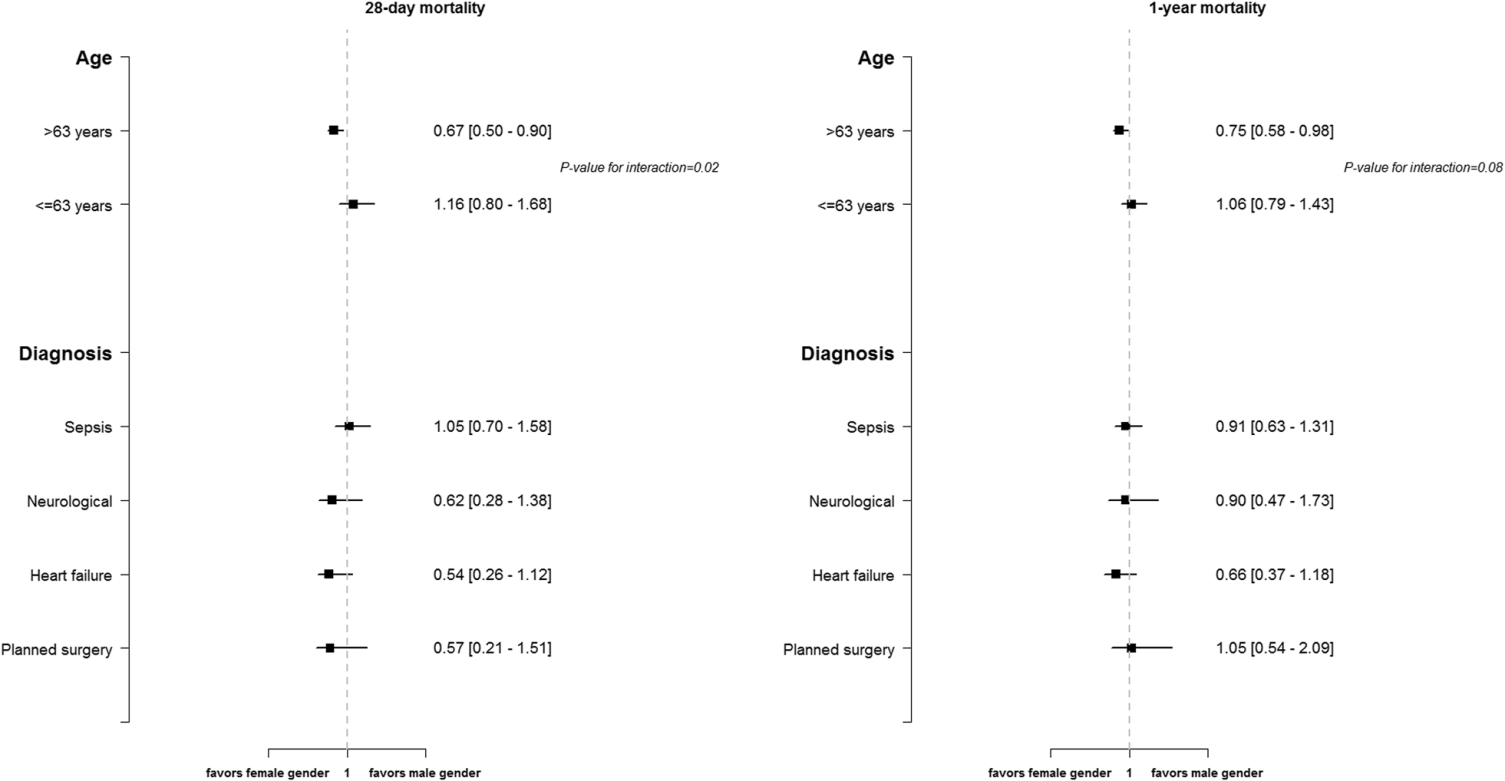
Subgroup analyses for age and diagnosis group at ICU admission

As depicted in Fig. 4, consistent results with similar survivals of women compared to men at both 1 year and 28 days after ICU admission were found, independently from the diagnosis at ICU admission. Notably, we observed a trend toward reduced hazard of death for older women compared to older men, in particular at 28 days after admission, as shown in Additional file 1: Figure 2.

## Discussion

Few studies analyzed gender-related differences in outcome of critically ill patients and found inconsistent results. This study aimed to test the hypothesis that gender is independently associated with long-term survival in a large, prospective, multi-centric cohort of critically ill patients. We found that women have similar long-term survival after ICU admission, compared to men.

First, the population of our study consisted of critically ill patients with relevant ICU mortality, in line with previous studies. Our study showed that women and men have some relevant differences in ICU referral diagnosis and comorbidities, as previously described and long known [11, 12]. However, and even more notably, women and men were unexpectedly similar regarding demographic characteristics, clinical presentation, disease severity, and ICU outcomes. Despite these similarities in baseline characteristics and outcome, and the effort to include all consecutive patients fulfilling inclusion criteria, we observed that only one of three patients included in our study was a woman. The reason for this imbalance is unknown but will require further investigations. We might anticipate that a combination of several factors, including gender-related variations in clinical presentation of disease, diverging patients’ preferences, and variable attitude of the treating teams, may have led to this imbalanced rate of ICU admissions.

Second, we observed consistently similar survival in women and men at both 28 days and 1 year after ICU admission. Survival remained similar after multivariable adjustment for comorbidities and disease severity and also in the propensity score-matched cohort to obviate difference in baseline characteristics. The impact of gender on prognosis has been thoroughly assessed in cardiology [13–24] and psychiatry [25–31], whereas most previous studies with critically ill patients analyzed the population as a whole, with little regard to gender-related differences and found inconsistent results [6]. To the best of our knowledge, the present study is the first to assess gender-related differences on long-term outcome of critically ill patients. Our observations are of great interest, since despite potential differences in genetic, hormonal, and immunological factors—as shown in other conditions [32, 33]—outcomes remain very similar for critically ill patients. Of course, other “non-biological” factors including socioeconomical differences may also have contributed or counterbalanced biological differences. However, a previous study from the FROG-ICU cohort showed a negligible impact of socioeconomic status on survival [32]. Furthermore, despite that a lacking social security coverage was considered an exclusion criterion, only one patient was excluded from the FROG-ICU cohort for this reason. Further research is needed to explore and distinguish biological from socioeconomical components affecting the long-term outcome of women and men.

From a more clinical point of view, our data support the fact that despite that the gender has been included in several widely used prognostic scores in other fields of medicine [33], none of the commonly used ICU prognostic scores, such as the Acute Physiology and Chronic Health Evaluation (APACHE) II, Simplified Acute Physiology Score (SAPS) II, Multiple Organ Dysfunction Score (MODS), and Sequential Organ Failure Assessment (SOFA), contain gender parameters, which is likely reasonable. Similar outcome between women and men may support the current practice of consistent treatment of the critical illness independently from the gender or, in light of the still severe prognosis of several diseases (i.e., septic shock, cardiogenic shock), a call for the implementation of precision medicine, with regard to differences beyond the gender. Nevertheless, gender-specific admission rates should be viewed as a potential risk of bias since only one-third of patients recruited in FROG-ICU were female. The male–female ratio in FROG-ICU was slightly more pronounced compared to other studies (Additional file 1: Table 1). If female gender could possibly bias, clinicians against admitting patients to the ICU a “protective” effect of female sex may have been confounded.

Third, our subgroup analysis showed a trend toward a survival benefit for women among elderly subjects. Very notably, older—and not younger—women seem to have a survival benefit compared to men. This observation challenges the current concept of the beneficial effect of estrogens on the incidence of several diseases and survival. This age-dependent differential impact of gender on the survival of critically ill patients is a novel finding, and the reasons remain to be elucidated.

We acknowledge that this study suffers from several limits. First, the observational nature of the data hinders confirmation of causality. Secondly, our data lack to account for variables with a substantial impact on the reported results, (i.e., socioeconomic status, patient compliance, and overall behavior (e.g., readiness to assume risk) after hospital discharge. Finally, the cause of death after ICU discharge was not registered in the FROG-ICU study.

## Conclusion

Women constituted one-third of the population of critically ill patients and had similar survival at 28 days and 1 year after ICU admission, independently from comorbidities and disease severity in a large, prospective, multi-centric cohort study. Older—and not younger—women may have a survival benefit compared to men. Overall, further research is needed focusing on the outcome before ICU admission, assessment of outcome of pre-hospital patient care targeting ICU referral, and disease prevalence and severity with respect to gender.

## Additional file

**Additional file 1.** All figures and tables are originals provided by the statistician (Elodie Féliot).
