# Supplementary material for: Gender and survival of critically ill patients: results from the FROG-ICU study
Source: Ann Intensive Care. 2019 Mar 29;9:43. doi: 10.1186/s13613-019-0514-y (PMC6441070; doi:10.1186/s13613-019-0514-y)

**Supplementary material**

**Supplementary Table 1**

| **Study** | **Country** | **Sample size** | **Ratio men/women (%)** | **Identifier** |
| --- | --- | --- | --- | --- |
| *FROG-ICU* | *France/Belgium* | 2’087 | *65 / 35* | *29347987* |
| Reinikeinen et al., 2005 | Finland | 24’341 | 62 / 38 | 16045660 |
| Garland et al., 2013 | Canada | 41’833 | 60 / 40 | 24079640 |
| Samuelsson et al., 2015 | Sweden | 127’254 | 57 / 43 | 25887421 |
| Laake et al., 2010 | Norway | N/A | 57 / 43 | 19930244 |
| ICNARC (UK), 2017-18 | United Kingdom | 211’612 | 55 / 45 | https://onlinereports.icnarc.org |
| Seymour et al., 2016 | USA | 706’399 | 54 /46 | 26903335 |

*ICNARC, intensive care national audit & research centre.*

**Supplementary Figure 1: Variables at inclusion for propensity score and representation of the standardized difference in percentage.**

**
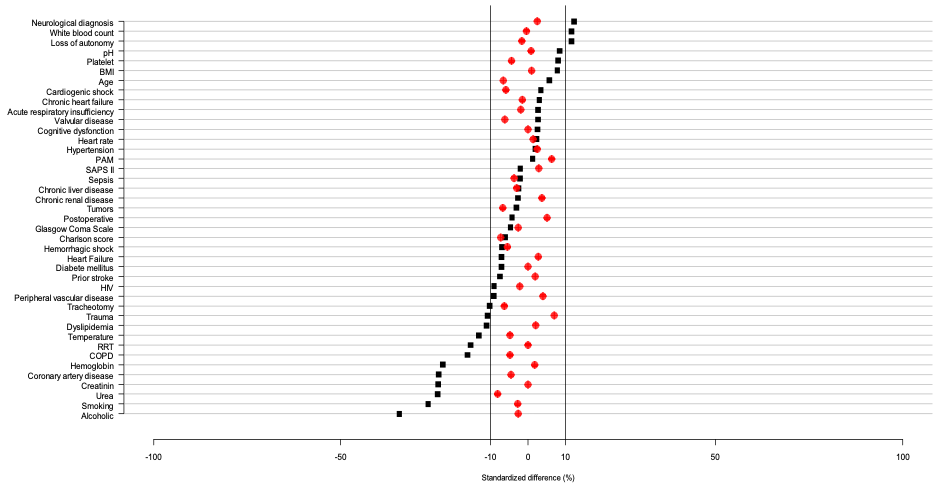
**

Black dots represent standardized differences in percentage before matching, whereas red dots are standardized differences in percentage after matching. After matching, all differences are between +/- 10%, indicating good balance.

**Supplementary Figure 2: Kaplan-Meier survival curve of 28-day and 1-year.**


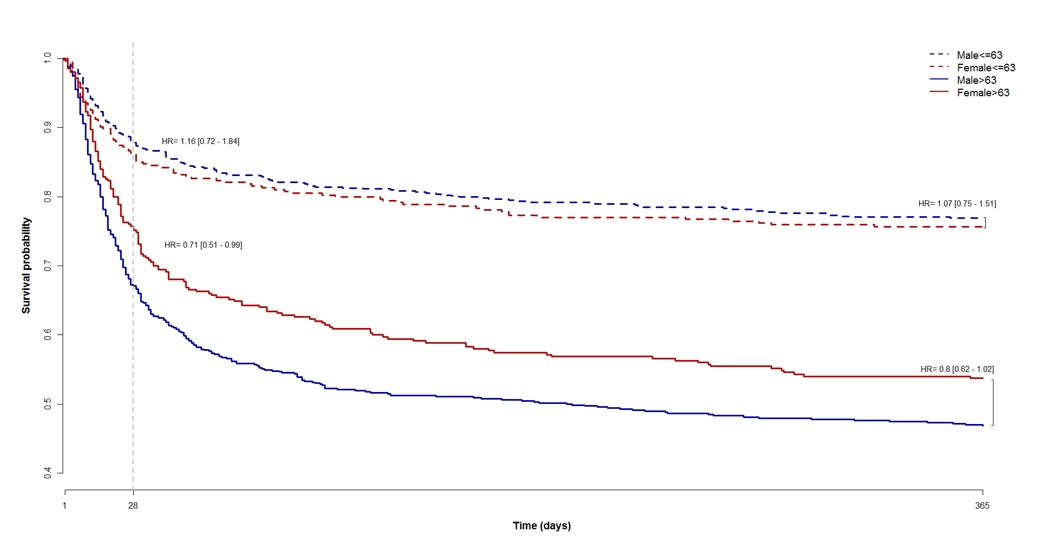

Supplement: Supplementary file 1 — Additional file 1. All figures and tables are originals provided by the statistician (Elodie Féliot). [file 13613_2019_514_MOESM1_ESM.docx]
